# MiR-25802: a potential target for treating Alzheimer’s disease by regulating neuroinflammation

**DOI:** 10.3389/fimmu.2024.1524432

**Published:** 2024-12-20

**Authors:** Kaiyue Zhao, Zixuan Li, Li Zeng, Zhongdi Cai, Rui Liu

**Affiliations:** Institute of Medicinal Biotechnology, Chinese Academy of Medical Sciences and Peking Union Medical College, Beijing, China

**Keywords:** Alzheimer’s disease, microRNA-25802, miRNA-based therapy, neuroinflammation, microglia

## 1 Introduction

Alzheimer’s disease (AD) holds global significance as a neurodegenerative disorder with a complex etiology still partly understood. Recent advancements in genetic and molecular research have underscored the intricate interplay of cellular pathways and gene networks, alongside feedforward and feedback regulatory mechanisms, which may differentially impact various pathogenic phenotypes and cellular stages of AD (1). Besides the strategies of neurotransmitter modulator interference and amyloid beta peptide (Aβ)-targeted plaque clearance, neuroinflammatory inhibitors have been widely explored as potential therapeutic approaches for AD, targeting toll-like receptors (TLRs), nuclear factor kappa-light-chain-enhancer of activated B cells (NF-κB), NOD-, LRR- and pyrin domain-containing protein 3 (NLRP3) inflammasome, among others (2–5). Notably, microglia play a pivotal role in clearing Aβ and damaged neuronal cells, crucial for maintaining the balance between pro- and anti-inflammatory processes during neuroinflammation. Their close association with tau phosphorylation, synapse loss, and cognitive decline makes these pathological processes central to AD research (6–11).

**Figure 1:**
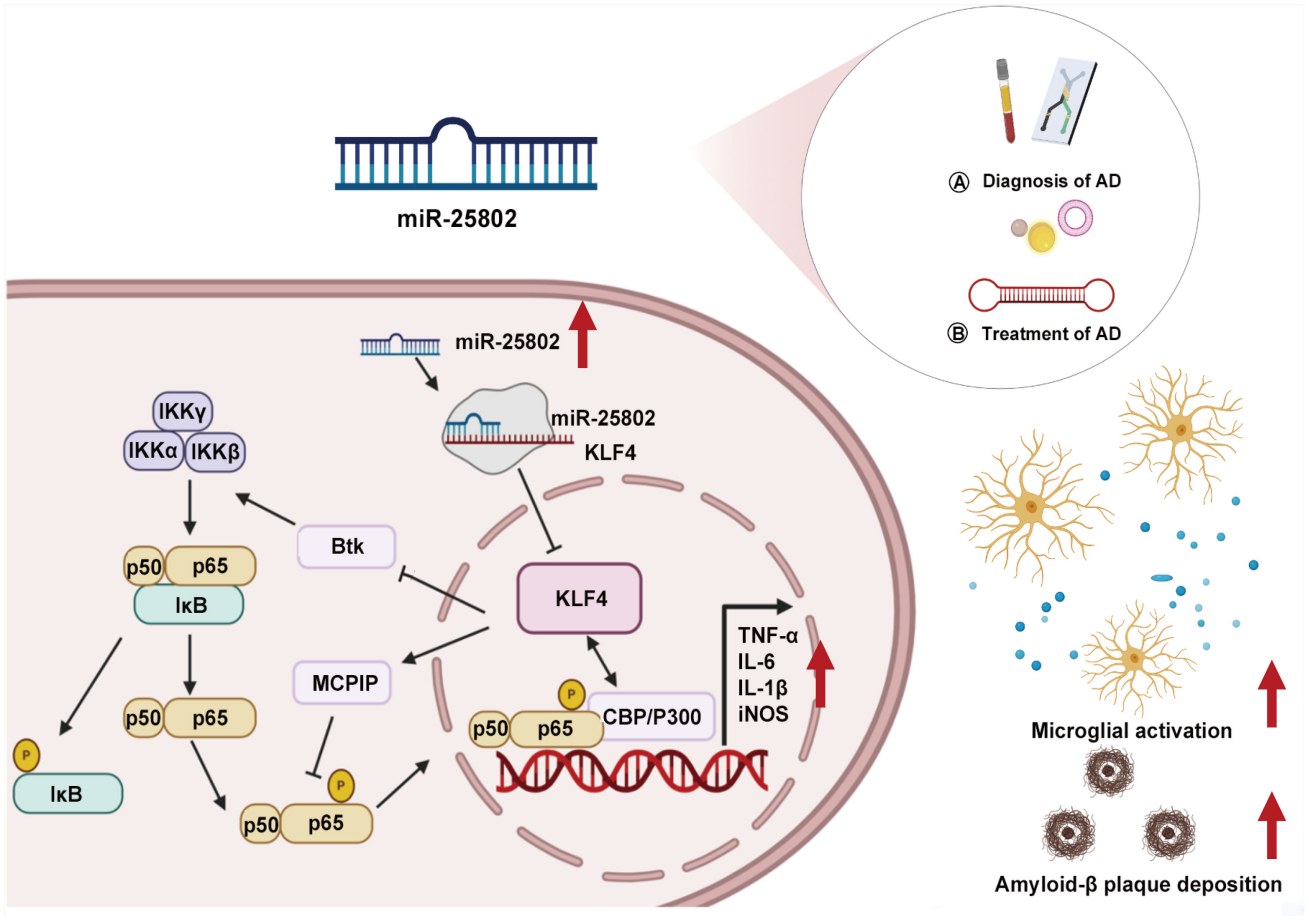
Role of miR-25802 in AD neuroinflammatory pathology. AD, Alzheimer’s disease; Btk, Bruton tyrosine kinase; CBP/P300, CREB-binding protein/p300; IKKα, IκB kinase α; IKKβ, IκB kinase β; IKKγ, IκB kinase γ; IL-1β, Interleukin-1β; IL-6, Interleukin-6; iNOS, Inducible nitric oxide synthase; IκB, Inhibitor of nuclear factor kappa B; KLF4, Kruppel-like factor 4; MCPIP, Monocyte chemotactic protein-induced protein; TNFα, Tumor necrosis factor-alpha.

MicroRNA (miRNA) modulation of neuroinflammation emerges as a critical regulator in maintaining microglial homeostasis by altering immunoinflammatory-related gene and protein expression. Dysregulation of this process can induce a variety of AD-associated pathological processes, including Aβ metabolism (12, 13), tau phosphorylation (14, 15), neuronal damage (16, 17), and synapse dysfunction (18, 19). The full scope of miRNA mechanisms in regulating neuroinflammation and related disorders remains under exploration. Our research highlights the crucial influence of a newly identified miRNA, miR-25802, on pathological neuroinflammation in AD (20–23). This discussion delves into the role of miR-25802 in AD pathology and its potential as a biomarker and target for future miRNA-based AD therapies (**Figure 1**).

## 2 The role of miR-25802 in microglial phenotypic transformation in AD

The novel base sequences miR-25802 were identified in the AD mouse brain utilizing high-throughput sequencing (20–23). miR-25802 expression is conserved across species and relies on the canonical miRNA enzymes Dicer and Drosha for biogenesis. Notably, significant upregulation of miR-25802 in the plasma of AD patients hints at potential diagnostic utility, and its upregulation in brain regions associated with learning and memory across various AD mouse models suggests a crucial role in AD pathology. The peak upregulation of miR-25802 occurs in AD models from 5 to 7 months, which aligns with the early microglial activation in AD (24).

Subsequent study addresses the functional implications of miR-25802 in AD-related neuroinflammation. First, miR-25802 promotes the activation of pro-inflammatory microglial cells, leading to increased release of pro-inflammatory cytokines such as tumor necrosis factor-alpha (TNF-α), interleukin-6 (IL-6), inducible nitric oxide synthase (iNOS), and interleukin-1-beta (IL-1β), while the inhibition of miR-25802 shifts microglia toward an anti-inflammatory phenotype, characterized by M2 markers such as arginase 1 (Arg1), macrophage mannose receptor 1(CD206), interleukin 4 (IL-4), and transforming growth factor beta (TGF-β), ultimately alleviating the inflammatory response. Second, *in vivo* functional evidence underscored the significant impact of modulating miR-25802 expression on AD pathology. Overexpression of miR-25802 via adeno-associated virus type 9 (AAV9) in 5×familial AD (5×FAD) mice exacerbated spatial learning and memory abilities, Aβ deposition, and microglial activation. Conversely, inhibiting miR-25802 improved cognitive impairment, reduced Aβ deposition, and attenuated microglial activation in AD mice. These effects of miR-25802 depend on its regulation of Kruppel-like factor 4 (KLF4), a direct target of miR-25802 involved in microglial M1/M2 phenotype conversion and neuroinflammatory responses.

## 3 miR-25802-mediated inflammatory signaling pathways in AD

Using bioinformatics, a dual-luciferase reporter assay, gain- and loss-of-function experiments, quantitative real-time polymerase chain reaction (qRT-PCR), and Western blot analyses, KLF4 was identified as a mediator of the effect of miR-25802 on microglia-mediated neuroinflammation in AD, both *in vitro* and *in vivo*. Overexpression or inhibition of miR-25802 post-transcriptionally regulated KLF4 expression. Consistently, KLF4 overexpression or silencing in 5×FAD mouse brain reversed the pathological characteristics associated with miR-25802 upregulation or inhibition, including cognitive impairment, Aβ deposition, microglial activation, and inflammatory reactions. KLF4 is a multifunctional transcription factor known to regulate inflammation in the peripheral tissue (25–27). Reports indicate that KLF4 suppresses the polarization of pro-inflammatory M1 macrophages while promoting anti-inflammatory M2 polarization (25, 28, 29). In line with these findings, silencing or overexpression of KLF4 had enhanced or inhibitory effects on the NF-κB cascades. By inhibiting KLF4 in microglia, miR-25802 encouraged microglial differentiation into the pro-inflammatory M1 phenotype, leading to excessive activation of the NF-κB signaling pathway and exacerbating the inflammatory response (20).

NF-κB plays a central role in microglial activation and inflammatory factor release in the brain of AD patients, mediating the downstream neuroinflammatory response triggered by Aβ deposition (30–32). Interestingly, miR-25802 has a genetic affinity with let-7a-2-3p, a member of the let-7 family identified with protective effects in immunoinflammatory disorders like stroke, AD, and multiple sclerosis. Upregulation of let-7 in macrophages promotes an anti-inflammatory phenotype by reducing expression of the transcription factor CCAAT/enhancer binding protein delta (C/ebp-δ) (33). Other inflammation-related targets of let-7 include *IL6* and toll-like receptor 4 (*Tlr4)* (34, 35). NF-κB inhibits let-7-mediated anti-inflammatory actions via inflammatory feedforward loops involving RNA-binding protein Lin28 or IL-6 signaling (36, 37). Combining gene sequence potential analysis with experimental evidence, the involvement of miR-25802/KLF4/NF-κB signaling in microglia-mediated neuroinflammation in AD has been established.

## 4 Clinical transformation prospects and strategies based on miR-25802 modulation

Recent studies have substantiated significant dysregulation of miRNA expression in the body fluids of AD patients (38, 39), which can be predictive of mild cognitive impairment (40), indicating its potential as a promising biomarker for early AD diagnosis. Notably, the emergence of biological miRNA sensing platforms has opened a new avenue for early AD detection, enabling real-time, sensitive detection of miRNAs in body fluids through electrochemical, fluorescence, and surface plasmon resonance biosensing technologies (41, 42). Our previous studies have revealed remarkable downregulations of miR-200a-3p (43) and miR-148a-3p (17) in the blood of AD patients, both playing protective roles in AD. As potential biomarkers, miR-200a-3p and miR-148a-3p have been successfully incorporated into biosensors to facilitate early detection of AD (44–46). *In vivo* models hypothesize the abnormal upregulation of miR-25802 contributes to neuroinflammation and cognitive deficits in the early stages of AD (20–23). Receiver operation curve analysis in the plasma of AD patients shows that miR-25802 expression levels have high diagnostic efficacy for cognitive dysfunction, suggesting its potential as an AD biomarker. Given the strong correlation between miR-25802 and AD progression, the early development of miR-25802-based diagnostic devices for AD is desirable.

Regarding miRNA-based treatment, preclinical studies have achieved precise gene expression regulation and cognitive improvement through the introduction of exogenous miRNAs using various miRNA delivery approaches, including viral vectors (12), liposomes (47), nanoparticles (48, 49), and exosomes (50). Among these, viral delivery systems, particularly due to their high efficiency in facilitating cellular RNA uptake, have attracted significant attention (51). For example, adeno-associated virus (AAV) type 5 serves as a delivery vector for AMT-130, a miRNA-based therapy currently in phase 2 clinical trials targeting Huntington’s disease (52, 53). Additionally, recombinant AAV9, a highly effective platform for central nervous system delivery, has been approved by the FDA for gene therapy in spinal muscular atrophy (54).

Importantly, our findings demonstrate that lateral ventricular injection of AAV9-encapsulated miR-25802 sponges in 5×FAD mice improved learning and memory abilities (20–23). Conversely, AAV9-coated KLF4 shRNA functioned to suppress the enhancement of learning and memory induced by miR-25802 sponges. This suggests that regulating miR-25802 expression or targeting its associated signaling pathways through AAV9 delivery might offer novel therapeutic approaches for AD, potentially counteracting disease progression and ameliorating cognitive deficits. Besides, the primary challenge in AD treatment remains the development of ncRNA-based therapies capable of effectively crossing the blood-brain barrier (55). Non-viral vectors have been investigated for miRNA-based treatment, particularly by packaging negatively charged miRNAs into liposome nanoparticles. This approach not only protects miRNAs from degradation by endogenous nucleases but also enables cell type-specific targeted delivery (56–58), presenting new opportunities for the development of suitable brain delivery systems for miR-25802.

## 5 Discussion

Microglia-driven neuroinflammation is implicated in both the initiation and progression of AD. Microglia activation serves as a defense mechanism for the brain against detrimental pathogens. However, this activation also incites inflammatory reactions that exacerbate the brain injury. Intriguingly, studies have predicted that more than 50% of mRNAs host miRNAs, allowing them to regulate an array of biological processes, encompassing oxidative stress, inflammation, and apoptosis (59). Specific miRNAs have emerged as potential mediators of neuroinflammation. For instance, miR-155 and miR-223 bind to TLRs to modulate the inflammatory response and influence the production of inflammatory mediators by sponging off suppressors of cytokine signaling in transgenic AD mouse models (60–62). Additionally, miRNAs like miR-146a, miR-873, and miR-34a are involved in neuropathology and neuroinflammation, regulated by NF-ĸB-associated signaling (63–65). Our research indicates that miR-25802, highly expressed in the brain and microglia across various AD models, is a functional RNA molecule devoid of coding capabilities but capable of regulating KLF4 expression and function through NF-κB signaling in neuroinflammation. The let-7 family, consisting of 8–12 members, has been reported to be dysregulated in AD compared to normal controls (66). miR-25802 exhibits high sequence similarity to the let-7 family, whose meta-analysis as biomarkers for early AD detection aligns with our identification of miR-25802 as an inflamma-miRNA based on microglial regulation in AD pathology.

miRNAs may emerge as therapeutic interventions for AD in the future. Currently, ncRNA-based therapies have been developed for complex, intractable diseases such as cardiovascular diseases and cancers (67, 68). In our prior studies, miR-25802 mimics, inhibitors, and sponges were designed based on functional modifications or complementary sequences. miR-25802 mimics, derived from exogenous synthesis and composed of small double-stranded RNA molecules, exacerbated microglial activation via aberrant regulation of the KLF4/NF-κB pathways both *in vitro* and *in vivo*. Conversely, miR-25802 inhibitors and sponges shielded anti-inflammatory microglial conversion by restoring KLF4/NF-κB inflammatory signaling *in vitro* and *in vivo*, respectively. Furthermore, intracranial injection of these mimics of miR-25802 into wild-type or 5×FAD mice resulted in no significant systemic discomfort or exercise capacity alterations during the one-month observation period. These findings suggest the miR-25802/KLF4/NF-κB pathway as a viable therapeutic target for AD.

## 6 Conclusions

Our opinion delves into the biological function, underlying mechanisms, and potential clinical application of miR-25802, an innovative non-coding RNA sequence, within the context of AD. miR-25802 orchestrates the KLF4/NF-κB pathway in microglia-mediated neuroinflammation, facilitating cognitive impairments and Aβ toxicity, highlighting the potential of targeting miR-25802 and its regulatory network as an emerging frontier in AD treatment. Future research on miR-25802 will be further advanced through potential interdisciplinary collaborations, including neuroscience, bioengineering, and clinical medicine. Initially, identifying the drivers of miR-25802 upregulation in AD and other specific mRNA targets using tissue-specific knockout technologies will be crucial. Additionally, clinical research necessitates exploring the diagnostic and differential diagnostic implications of the miR-25802/KLF4 axis across a broader patient cohort. Furthermore, most miRNA-centric diagnostic tools and therapeutic interventions for AD are still in their initial stage. Regarding miR-25802, biological macromolecular inhibitors, employing oligonucleotide analogues as scaffolds, may afford benefits such as high selectivity and efficiency; however, they also pose challenges like immunogenicity and multiple conformations. Alternatively, the development of small molecule inhibitors targeting miR-25802 and its signaling pathway boasts its own strengths, including robust cell membrane penetration, resistance to intracellular enzymatic degradation, and reduced synthesis costs. In summary, achieving a profound understanding of the mechanism of miR-25802 in AD, coupled with the creation of more effective delivery systems or targeted small molecule drugs, will pave the way for translating miR-25802 research from the laboratory to clinical practice.

## References

[1] Murdock MH Tsai LH. Insights into Alzheimer’s disease from single-cell genomic approaches. Nat Neurosci. (2023) 26:181–95. doi: 10.1038/s41593-022-01222-2 PMC10155598 36593328

[2] Cummings JL Osse AML Kinney JW. Alzheimer’s disease: novel targets and investigational drugs for disease modification. Drugs. (2023) 83:1387–408. doi: 10.1007/s40265-023-01938-w PMC10582128 37728864

[3] Rangasamy SB Jana M Roy A Corbett GT Kundu M Chandra S. Selective disruption of TLR2-MyD88 interaction inhibits inflammation and attenuates Alzheimer’s pathology. J Clin Invest. (2018) 128:4297–312. doi: 10.1172/JCI96209 PMC6159992 29990310

[4] Sun E Motolani A Campos L Lu T. The pivotal role of NF-kB in the pathogenesis and therapeutics of alzheimer’s disease. Int J Mol Sci. (2022) 23:8972. doi: 10.3390/ijms23168972 36012242 PMC9408758

[5] McManus RM Latz E. NLRP3 inflammasome signaling in Alzheimer’s disease. Neuropharmacology. (2024) 252:109941. doi: 10.1016/j.neuropharm.2024.109941 38565393

[6] Arranz AM De Strooper B. The role of astroglia in Alzheimer’s disease: pathophysiology and clinical implications. Lancet Neurol. (2019) 18:406–14. doi: 10.1016/S1474-4422(18)30490-3.E 30795987

[7] Ralvenius WT Andresen JL Huston MM Penney J Bonner JM Fenton OS. Nanoparticle-Mediated Delivery of Anti-PU.1 siRNA via Localized Intracisternal Administration Reduces Neuroinflammation. Adv Mater. (2024) 36:e2309225. doi: 10.1002/adma.202309225 38018280

[8] Rego S Sanchez G Da Mesquita S. Current views on meningeal lymphatics and immunity in aging and Alzheimer’s disease. Mol Neurodegener. (2023) 18:55. doi: 10.1186/s13024-023-00645-0 37580702 PMC10424377

[9] Agarwal D Sandor C Volpato V Caffrey TM Monzón-Sandoval J Bowden R. A single-cell atlas of the human substantia nigra reveals cell-specific pathways associated with neurological disorders. Nat Commun. (2020) 11:4183. doi: 10.1038/s41467-020-17876-0 32826893 PMC7442652

[10] Chen Y Colonna M. Microglia in Alzheimer’s disease at single-cell level. Are there common patterns in humans and mice? J Exp Med. (2021) 218:e20202717. doi: 10.1084/jem.20202717 34292312 PMC8302448

[11] Li X Li C Zhang W Wang Y Qian P Huang H. Inflammation and aging: signaling pathways and intervention therapies. Signal Transduct Target Ther. (2023) 8:239. doi: 10.1038/s41392-023-01502-8 37291105 PMC10248351

[12] Yin Z Herron S Silveira S Kleemann K Gauthier C Mallah D. Identification of a protective microglial state mediated by miR-155 and interferon-γ signaling in a mouse model of Alzheimer’s disease. Nat Neurosci. (2023) 26:1196–207. doi: 10.1038/s41593-023-01355-y PMC10619638 37291336

[13] Sun T Zhao K Liu M Cai Z Zeng L Zhang J. miR-30a-5p induces Aβ production via inhibiting the nonamyloidogenic pathway in Alzheimer’s disease. Pharmacol Res. (2022) 178:106153. doi: 10.1016/j.phrs.2022.106153 35257899

[14] Kodama L Guzman E Etchegaray JI Li Y Sayed FA Zhou L. Microglial microRNAs mediate sex-specific responses to tau pathology. Nat Neurosci. (2020) 23:167–71. doi: 10.1038/s41593-019-0560-7 PMC7394069 31873194

[15] Jiang H Liu J Guo S Zeng L Cai Z Zhang J. miR-23b-3p rescues cognition in Alzheimer’s disease by reducing tau phosphorylation and apoptosis via GSK-3β signaling pathways. Mol Ther Nucleic Acids. (2022) 28:539–57. doi: 10.1016/j.omtn.2022.04.008 PMC9092887 35592504

[16] Wei H Zhu Z Xu Y Lin L Chen Q Liu Y. Microglia-derived exosomes selective sorted by YB-1 alleviate nerve damage and cognitive outcome in Alzheimer’s disease. J Transl Med. (2024) 22:466. doi: 10.1186/s12967-024-05256-x 38755651 PMC11100039

[17] Zeng L Jiang H Ashraf GM Liu J Wang L Zhao K. Implications of miR-148a-3p/p35/PTEN signaling in tau hyperphosphorylation and autoregulatory feedforward of Akt/CREB in Alzheimer’s disease. Mol Ther Nucleic Acids. (2022) 27:256–75. doi: 10.1016/j.omtn.2021.11.019 PMC8714918 35024240

[18] Li X Chen SC Ip JPK. Diverse and composite roles of miRNA in non-neuronal cells and neuronal synapses in Alzheimer’s disease. Biomolecules. (2022) 12:1505. doi: 10.3390/biom12101505 36291714 PMC9599315

[19] Dalal S Ramirez-Gomez J Sharma B Devara D Kumar S. MicroRNAs and synapse turnover in Alzheimer’s disease. Ageing Res Rev. (2024) 99:102377. doi: 10.1016/j.arr.2024.102377 38871301

[20] Zhao K Liu J Sun T Zeng L Cai Z Li Z. The miR-25802/KLF4/NF-κB signaling axis regulates microglia-mediated neuroinflammation in Alzheimer’s disease. Brain Behav Immun. (2024) 118:31–48. doi: 10.1016/j.bbi.2024.02.016 38360375

[21] Liu R Li Z Zhao K Liu M Zeng L Sun T. Biomarker miR-25802 cluster for inflammation-related diseases and application of biomarker miR-25802 cluster (2022). Available online at: https://patentscope2.wipo.int/search/zh/detail.jsf?docId=CN397428498 (accessed December 14, 2024).

[22] Liu R Li Z Zhao K Liu M Zeng L. Biomarker Mir-25802 Cluster for inflammation-related diseases, and use thereof. PCT/CN2023/108782 W.O (2024). Available online at: https://patentscope.wipo.int/search/zh/detail.jsf?docId=WO2024012600 (accessed December 14, 2024).

[23] Liu R Li Z Zhao K Liu M Zeng L. Biomarker Mir-25802 Cluster for inflammation-related diseases, and use thereoff (2024). Available online at: https://patentcenter.uspto.gov/applications/18713459?application= (accessed December 14, 2024).

[24] Leng F Edison P. Neuroinflammation and microglial activation in Alzheimer disease: where do we go from here? Nat Rev Neurol. (2021) 17:157–72. doi: 10.1038/s41582-020-00435-y 33318676

[25] Kapoor N Niu J Saad Y Kumar S Sirakova T Becerra E. Transcription factors STAT6 and KLF4 implement macrophage polarization via the dual catalytic powers of MCPIP. J Immunol. (2015) 194:6011–23. doi: 10.4049/jimmunol.1402797 PMC4458412 25934862

[26] Ma C Xia R Yang S Liu L Zhang J Feng K. Formononetin attenuates atherosclerosis via regulating interaction between KLF4 and SRA in apoE-/- mice. Theranostics. (2020) 10:1090–106. doi: 10.7150/thno.38115 PMC6956811 31938053

[27] Cao Y Xiong J Guan X Yin S Chen J Yuan S. Paeoniflorin suppresses kidney inflammation by regulating macrophage polarization via KLF4-mediated mitophagy. Phytomedicine. (2023) 16:154901. doi: 10.1016/j.phymed.2023.154901 37247587

[28] Li Z Martin M Zhang J Huang HY Bai L Zhang J. Krüppel-like factor 4 regulation of cholesterol-25-hydroxylase and liver X receptor mitigates atherosclerosis susceptibility. Circulation. (2017) 136:1315–30. doi: 10.1161/CIRCULATIONAHA.117.027462 PMC5741092 28794002

[29] Wen Y Lu X Ren J Privratsky JR Yang B Rudemiller NP. KLF4 in macrophages attenuates TNFα-mediated kidney injury and fibrosis. J Am Soc Nephrol. (2019) 30:1925–38. doi: 10.1681/ASN.2019020111 PMC6779357 31337692

[30] Lian H Yang L Cole A Sun L Chiang AC Fowler SW. NFκB-activated astroglial release of complement C3 compromises neuronal morphology and function associated with Alzheimer’s disease. Neuron. (2015) 85:101–15. doi: 10.1016/j.neuron.2014.11.018 PMC4289109 25533482

[31] Sun J Zhang Y Kong Y Ye T Yu Q Kumaran Satyanarayanan S. Microbiota-derived metabolite Indoles induced aryl hydrocarbon receptor activation and inhibited neuroinflammation in APP/PS1 mice. Brain Behav Immun. (2022) 106:76–88. doi: 10.1016/j.bbi.2022.08.003 35961580

[32] Rahimifard M Maqbool F Moeini-Nodeh S Niaz K Abdollahi M Braidy N. Targeting the TLR4 signaling pathway by polyphenols: A novel therapeutic strategy for neuroinflammation. Ageing Res Rev. (2017) 36:11–9. doi: 10.1016/j.arr.2017.02.004 28235660

[33] Wang L Liu T Chen G Li Y Zhang S Mao L. Exosomal microRNA let-7-5p from Taenia pisiformisCysticercus Prompted Macrophage to M2 Polarization through Inhibiting the Expression of C/EBP δ. Microorganisms. (2021) 9:1403. doi: 10.3390/microorganisms9071403 34209741 PMC8307393

[34] Di T Yang Y Fu C Zhang Z Qin C Sai X. Let-7 mediated airway remodeling in chronic obstructive pulmonary disease via the regulation of IL-6. Eur J Clin Invest. (2021) 51:e13425. doi: 10.1111/eci.13425 33037614 PMC7988621

[35] Chen XM Splinter PL O’Hara SP LaRusso NF. A cellular micro-RNA, let-7i, regulates Toll-like receptor 4 expression and contributes to cholangiocyte immune responses against Cryptosporidium parvum infection. J Biol Chem. (2007) 282:28929–38. doi: 10.1074/jbc.M702633200 PMC2194650 17660297

[36] Kang M Lee KH Lee HS Jeong CW Ku JH Kim HH. Concurrent treatment with simvastatin and NF-κB inhibitor in human castration-resistant prostate cancer cells exerts synergistic anti-cancer effects via control of the NF-κB/LIN28/let-7 miRNA signaling pathway. PloS One. (2017) 12:e0184644. doi: 10.1371/journal.pone.0184644 28910332 PMC5599006

[37] Cao Q Li YY He WF Zhang ZZ Zhou Q Liu X. Interplay between microRNAs and the STAT3 signaling pathway in human cancers. Physiol Genomics. (2013) 45:1206–14. doi: 10.1152/physiolgenomics.00122.2013 24192393

[38] Swarbrick S Wragg N Ghosh S Stolzing A. Systematic review of miRNA as biomarkers in Alzheimer’s disease. Mol Neurobiol. (2019) 56:6156–67. doi: 10.1007/s12035-019-1500-y PMC6682547 30734227

[39] Han SW Pyun JM Bice PJ Bennett DA Saykin AJ Kim SY. miR-129-5p as a biomarker for pathology and cognitive decline in Alzheimer’s disease. Alzheimers Res Ther. (2024) 16:5. doi: 10.1186/s13195-023-01366-8 38195609 PMC10775662

[40] Kumar A Su Y Sharma M Singh S Kim S Peavey JJ. MicroRNA expression in extracellular vesicles as a novel blood-based biomarker for Alzheimer’s disease. Alzheimers Dement. (2023) 19:4952–66. doi: 10.1002/alz.13055 PMC11663460 37071449

[41] Pishbin E Sadri F Dehghan A Kiani MJ Hashemi N Zare I. Recent advances in isolation and detection of exosomal microRNAs related to Alzheimer’s disease. Environ Res. (2023) 227:115705. doi: 10.1016/j.envres.2023.115705 36958383

[42] Pereira RL Oliveira D Pêgo AP Santos SD Moreira FTC. Electrochemical miRNA-34a-based biosensor for the diagnosis of Alzheimer’s disease. Bioelectrochemistry. (2023) 154:108553. doi: 10.1016/j.bioelechem.2023.108553 37672968

[43] Wang L Liu J Wang Q Jiang H Zeng L Li Z. MicroRNA-200a-3p mediates neuroprotection in alzheimer-related deficits and attenuates amyloid-beta overproduction and tau hyperphosphorylation via coregulating BACE1 and PRKACB. Front Pharmacol. (2019) 10:806. doi: 10.3389/fphar.2019.00806 31379578 PMC6658613

[44] Na HK Wi JS Son HY Ok JG Huh YM Lee TG. Discrimination of single nucleotide mismatches using a scalable, flexible, and transparent three-dimensional nanostructure-based plasmonic miRNA sensor with high sensitivity. Biosens Bioelectron. (2018) 113:39–45. doi: 10.1016/j.bios.2018.04.033 29727750

[45] Wang Y Howes PD Kim E Spicer CD Thomas MR Lin Y. Duplex-specific nuclease-amplified detection of microRNA using compact quantum dot-DNA conjugates. ACS Appl Mater Interf. (2018) 10:28290–300. doi: 10.1021/acsami.8b07250 PMC6141140 30113161

[46] Park JS Kim ST Kim SY Jo MG Choi MJ Kim MO. A novel kit for early diagnosis of Alzheimer’s disease using a fluorescent nanoparticle imaging. Sci Rep. (2019) 9:13184. doi: 10.1038/s41598-019-49711-y 31515517 PMC6742761

[47] Su D Chen Z An X Yang J Yang J Wang X. MicroRNA-195 liposomes for therapy of Alzheimer’s disease. J Control Release. (2024) 365:583–601. doi: 10.1016/j.jconrel.2023.12.003 38048963

[48] Ouyang Q Liu K Zhu Q Deng H Le Y Ouyang W. Brain-penetration and neuron-targeting DNA nanoflowers co-delivering miR-124 and rutin for synergistic therapy of Alzheimer’s disease. Small. (2022) 18:e2107534. doi: 10.1002/smll.202107534 35182016

[49] Israel LL Sun T Braubach O Cox A Shatalova ES Rashid HM. [amp]]beta;-Amyloid targeting nanodrug for neuron-specific delivery of nucleic acids in Alzheimer’s disease mouse models. J Control Release. (2023) 361:636–58. doi: 10.1016/j.jconrel.2023.08.001 37544515

[50] Chen C Bao Y Xing L Jiang C Guo Y Tong S. Exosomes derived from M2 microglial cells modulated by 1070-nm light improve cognition in an Alzheimer’s disease mouse model. Adv Sci (Weinh). (2023) 10:e2304025. doi: 10.1002/advs.202304025 37702115 PMC10646245

[51] Gao J Gunasekar S Xia ZJ Shalin K Jiang C Chen H. Gene therapy for CNS disorders: modalities, delivery and translational challenges. Nat Rev Neurosci. (2024) 25:553–72. doi: 10.1038/s41583-024-00829-7 38898231

[52] Estevez-Fraga C Tabrizi SJ Wild EJ. Huntington’s disease clinical trials corner: March 2024. J Huntingtons Dis. (2024) 13:1–14. doi: 10.3233/JHD-240017 38489195 PMC11091610

[53] Estevez-Fraga C Tabrizi SJ Wild EJ. Huntington’s disease clinical trials corner: November 2022. J Huntingtons Dis. (2022) 11:351–67. doi: 10.3233/JHD-229006 36463457

[54] Mendell JR Al-Zaidy S Shell R Arnold WD Rodino-Klapac LR Prior TW. Single-dose gene-replacement therapy for spinal muscular atrophy. N Engl J Med. (2017) 377:1713–22. doi: 10.1056/NEJMoa1706198 29091557

[55] Min HS Kim HJ Naito M Ogura S Toh K Hayashi K. Systemic brain delivery of antisense oligonucleotides across the blood-brain barrier with a glucose-coated polymeric nanocarrier. Angew Chem Int Ed Engl. (2020) 59:8173–80. doi: 10.1002/anie.201914751 PMC7317551 31995252

[56] Roberts TC Langer R Wood MJA. Advances in oligonucleotide drug delivery. Nat Rev Drug Discovery. (2020) 19:673–94. doi: 10.1038/s41573-020-0075-7 PMC7419031 32782413

[57] Blanco E Shen H Ferrari M. Principles of nanoparticle design for overcoming biological barriers to drug delivery. Nat Biotechnol. (2015) 33:941–51. doi: 10.1038/nbt.3330 PMC4978509 26348965

[58] Lee SWL Paoletti C Campisi M Osaki T Adriani G Kamm RD. MicroRNA delivery through nanoparticles. J Control Release. (2019) 313:80–95. doi: 10.1016/j.jconrel.2019.10.007 31622695 PMC6900258

[59] Mushtaq G Greig NH Anwar F Zamzami MA Choudhry H Shaik MM. miRNAs as circulating biomarkers for Alzheimer’s disease and Parkinson’s disease. Med Chem. (2016) 12:217–25. doi: 10.2174/1573406411666151030112140 PMC6138249 26527155

[60] Fabbri M. TLRs as miRNA receptors. Cancer Res. (2012) 72:6333–7. doi: 10.1158/0008-5472.CAN-12-3229 23222301

[61] Guedes JR Custódia CM Silva RJ de Almeida LP Pedroso de Lima MC Cardoso AL. Early miR-155 upregulation contributes to neuroinflammation in Alzheimer’s disease triple transgenic mouse model. Hum Mol Genet. (2014) 23:6286–301. doi: 10.1093/hmg/ddu348 24990149

[62] Duan R Wang SY Wei B Deng Y Fu XX Gong PY. Angiotensin-(1-7) Analogue AVE0991 Modulates Astrocyte-Mediated Neuroinflammation via lncRNA SNHG14/miR-223-3p/NLRP3 Pathway and Offers Neuroprotection in a Transgenic Mouse Model of Alzheimer’s Disease. J Inflammation Res. (2021) 14:7007–19. doi: 10.2147/JIR.S343575 PMC8694579 34955647

[63] Nakano M Kubota K Kobayashi E Chikenji TS Saito Y Konari N. Bone marrow-derived mesenchymal stem cells improve cognitive impairment in an Alzheimer’s disease model by increasing the expression of microRNA-146a in hippocampus. Sci Rep. (2020) 10:10772. doi: 10.1038/s41598-020-67460-1 32612165 PMC7330036

[64] Liu X He F Pang R Zhao D Qiu W Shan K. Interleukin-17 (IL-17)-induced microRNA 873 (miR-873) contributes to the pathogenesis of experimental autoimmune encephalomyelitis by targeting A20 ubiquitin-editing enzyme. J Biol Chem. (2014) 289:28971–86. doi: 10.1074/jbc.M114.577429 PMC4200254 25183005

[65] Periyasamy P Thangaraj A Bendi VS Buch S. HIV-1 Tat-mediated microglial inflammation involves a novel miRNA-34a-NLRC5-NFκB signaling axis. Brain Behav Immun. (2019) 80:227–37. doi: 10.1016/j.bbi.2019.03.011 PMC6660398 30872089

[66] Cătană CS Marta MM Văleanu M Dican L Crişan CA. Human leukocyte antigen and microRNAs as key orchestrators of mild cognitive impairment and Alzheimer’s disease: A systematic review. Int J Mol Sci. (2024) 25:8544. doi: 10.3390/ijms25158544 39126112 PMC11312697

[67] Shah AM Giacca M. Small non-coding RNA therapeutics for cardiovascular disease. Eur Heart J. (2022) 43:4548–61. doi: 10.1093/eurheartj/ehac463 PMC9659475 36106499

[68] Coan M Haefliger S Ounzain S Johnson R. Targeting and engineering long non-coding RNAs for cancer therapy. Nat Rev Genet. (2024) 25:578–95. doi: 10.1038/s41576-024-00693-2 38424237

